# Draft Genome Sequence of *Bacillus thuringiensis* NBIN-866 with High Nematocidal Activity

**DOI:** 10.1128/genomeA.00429-14

**Published:** 2014-05-22

**Authors:** Xiaoyan Liu, Ronghua Zhou, Guiping Fu, Wei Zhang, Yong Min, Yuxi Tian, Daye Huang, Kaimei Wang, Zhongyi Wan, Jingwu Yao, Ziwen Yang

**Affiliations:** a National Biopesticide Engineering Technology Research Center, Hubei Biopesticide Engineering Research Center, Hubei Academy of Agricultural Sciences, Wuhan, China; b The Institute for the Control of Agrochemicals, Ministry of Agriculture, Beijing, China; c Department of Horticulture, Hubei Vocational College of Bio-Technology, Wuhan, China

## Abstract

*Bacillus thuringiensis* NBIN-866, a Gram-positive bacterium, was isolated from soil in China. We announce here the draft genome sequence of strain *B. thuringiensis* NBIN-866, which possesses highly nematocidal factors, such as proteins and small molecular peptides.

## GENOME ANNOUNCEMENT

*Bacillus thuringiensis* is a Gram-positive spore-forming soil bacterium with the ability to produce insecticidal crystal proteins (1–4). It shows toxicity to certain insect species (5, 6) and has been widely used as a biopesticide for controlling agricultural pests. *B. thuringiensis* can produce multiinsecticidal metabolites, like insecticide crystal proteins (7), cytotoxin (8), vegetative insecticidal protein (9), secret insecticidal protein (10), thuringiensin (11), zwittermicin A (12), Mtx-like toxin (13), Bin-like toxin (14), and more. The diversity of toxins is paralleled by diverse pesticidal activity.

Strain NBIN-866 was isolated from cotton field soil in China. Its 16S rRNA sequence has 99.0% similarity to that of *B. thuringiensis*. The genome sequencing of *B. thuringiensis* NBIN-866 was performed with a strategy involving Solexa paired-end sequencing technology. Two libraries containing 400-bp and 750-bp inserts were constructed. Sequencing was performed with the paired-end strategy of 502-bp reads to produce 645 Mb and 294 Mb of ﬁltered sequences, respectively, representing 170-fold coverage, with an Illumina Solexa Genome Analyzer (GA) IIx (Wuhan Yanxing Biotechnology Co., Ltd., Wuhan, China), and the reads were assembled into 203 contigs and 162 scaffolds using the Short Oligonucleotides Alignment Program (SOAP) *de novo* alignment tool (http://soap.genomics.org.cn/index.html#intro2). The gaps both within and between the scaffolds were ﬁlled through sequencing PCR products by primer walking, using an ABI 3730 capillary sequencer.

The genome of strain NBIN-866 consists of a 5,509,689-bp chromosome with a G+C content of 36.22%. The chromosome consists of 6,121 coding sequences (CDS), 4 rRNA operons, and 82 tRNAs. Genome annotation was performed with the NCBI Prokaryotic Genomes Automatic Annotation Pipeline (PGAAP), and the GenBank nonredundant (NR), Kyoto Encyclopedia of Genes and Genomes (KEGG) (15), and Clusters of Orthologous Groups (COG) (16) databases were employed for BLASTp identiﬁcation (17). Four gene clusters, covering 2.8% of the whole genome, were involved in the synthesis of antibiotics, such as butirosin, penicillin, tetracycline, and novobiocin. The insecticide crystal protein genes *cry1Ae* and *cry2Ac* were detected in NBIN-866.

### Nucleotide sequence accession number.

The complete sequence of *B. thuringiensis* NBIN-866 has been deposited in NCBI under the accession no. JEOF01000000.
